# Spotlight on Perfusion: Reshaping Oropharyngeal Squamous Cell Carcinoma Surgery

**DOI:** 10.7759/cureus.87480

**Published:** 2025-07-07

**Authors:** Fernando Dip, Guillermo Artero, Alberto Rancati, Javier I Ghiselli, Rene Aleman

**Affiliations:** 1 Department of General Surgery, University of Buenos Aires, Buenos Aires, ARG; 2 Heart, Vascular, and Thoracic Institute, Cleveland Clinic Florida, Weston, USA

**Keywords:** fluorescence guided surgery, head and neck surgery, indocyanine green, new surgical technologies, oncologic surgery

## Abstract

Oropharyngeal squamous cell carcinoma (OPSCC), commonly referred to as throat or tonsil cancer, is a type of head and neck malignancy arising from the base and posterior third of the tongue, tonsils, soft palate, and posterolateral pharyngeal walls. Standard treatment typically involves a single modality or a combination of surgery, radiotherapy, and/or chemotherapy. Despite favorable oncologic outcomes, chemoradiation is associated with considerable acute and long-term toxicities, including treatment-related mortality, debilitating dysphagia, fibrosis, severe xerostomia, and an increased risk of secondary malignancies. In this context, fluorescence-guided surgery (FGS) has emerged as a promising, minimally invasive intraoperative tool for OPSCC surgery. By leveraging indocyanine green (ICG), FGS offers enhanced visualization and real-time tissue differentiation, facilitating precise tumor resection and reducing the risk of surgical complications. This case presents an 83-year-old female patient who underwent radical OPSCC resection followed by complex reconstructive surgery utilizing a novel near-infrared FGS device.

## Introduction

Oropharyngeal squamous cell carcinoma (OPSCC) is among the most common malignant neoplasms of the head and neck, presenting significant challenges in surgical management. Over 90% of OPSCCs arise from the epithelial lining of the oropharynx and are histologically classified as squamous cell carcinomas [1]. These tumors are broadly categorized as human papillomavirus (HPV)-associated and non-HPV-associated subtypes. In the United States, the incidence of OPSCC continues to rise, particularly in HPV-mediated cases, a trend projected to persist beyond 2030 [2]. This shifting epidemiology has fueled ongoing debate regarding optimal therapeutic strategies. Standard treatment modalities, including surgery with or without adjuvant therapy or radiotherapy (RT) with or without chemotherapy, are endorsed by both the National Comprehensive Cancer Network and the American Society of Clinical Oncology (ASCO). However, these approaches are often associated with considerable morbidity [3]. Recent advances in magnification, optical imaging, and surgical technology have driven the integration of minimally invasive techniques aimed at mitigating the inadvertent complications related to resection and reconstruction in OPSCC management. Fluorescence-guided surgery (FGS) using indocyanine green (ICG) has demonstrated efficacy in real-time tumor-margin visualization, improving both margin clearance rates and progression-free survival in head and neck cancers [4]. Additionally, FGS enables intraoperative tissue perfusion assessment, supporting a nuanced approach that prioritizes oncologic clearance while preserving functional and aesthetic outcomes [5]. This case report presents the use of a novel near-infrared (NIR) perfusion monitoring device for FGS during OPSCC resection in an elderly patient with multiple comorbidities. The case highlights the clinical utility of this technology in enhancing surgical precision and optimizing outcomes.

## Case presentation

This is the case of an 83-year-old female patient who presented with a biopsy-confirmed OPSCC involving the right buccal mucosa. The patient’s past medical history included hypertension, managed with antihypertensive therapy, and type 2 diabetes mellitus, controlled with oral hypoglycemics. The patient indicated that she had a 40-year history of tobacco use. During the initial clinical evaluation, the lesion was observed 2 mm from the midline and extended approximately 1 cm posteriorly from the retromolar trigone. Initial measurements suggested a localized tumor with potential for focal invasion. Due to her advanced age and comorbidities, she was classified as high-risk during the preoperative evaluation, warranting detailed surgical planning and enhanced intraoperative monitoring. Laboratory workup was negligible. The patient underwent a diagnostic incisional biopsy under sedation. Approximately one week after said diagnostic intervention, the patient developed a cutaneous fistula at the lesion site, suggestive of either local disease progression or biopsy-related complication. This prompted expedited surgical intervention to prevent further deterioration. A multidisciplinary team formulated a plan for definitive oncologic resection, microvascular free flap reconstruction (FFR), and ipsilateral suprahyoid lymphadenectomy to manage potential regional lymphatic spread. The patient provided verbal and written consent prior to the intervention.

Surgical technique

**Figure 1:**
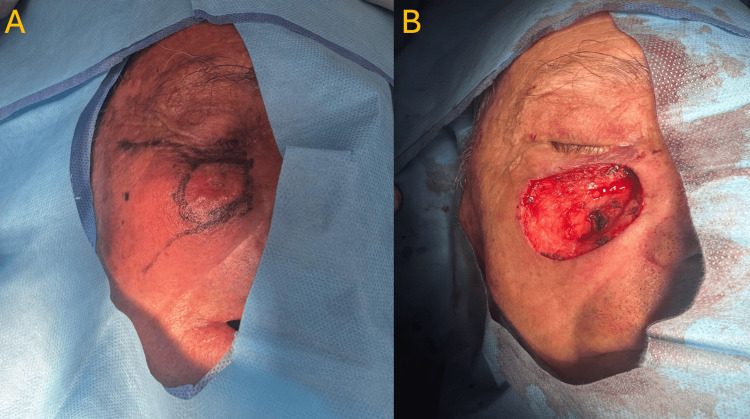
OPSCC resection (A) Location of the OPSCC prior to primary radical resection. (B) Post-radical resection of the OPSCC with negative margins. OPSCC: oropharyngeal squamous cell carcinoma

**Figure 2:**
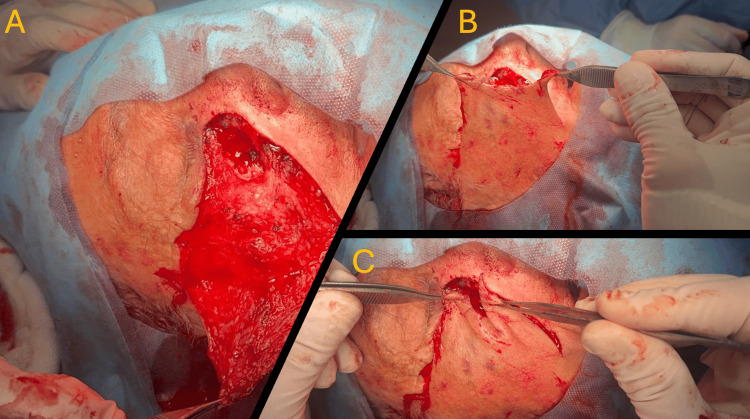
FFR (A-C) ALT flap, FFR transition. ALT: anterolateral thigh, FFR: free flap reconstruction

**Figure 3:**
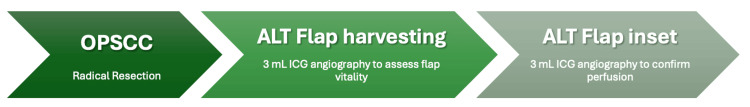
Timeline diagram Operative approach summarizing the times and dosages of ICG administered during the procedure to assess flap vitality prior to and post-inset. OPSCC: oropharyngeal squamous cell carcinoma, ALT: anterolateral thigh, ICG: indocyanine green

**Figure 4:**
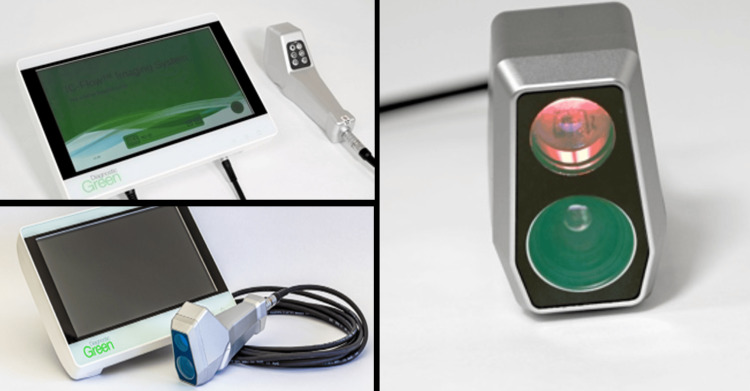
IC-Flow 2 device IC-Flow 2^TM^ device for indocyanine green NIR imaging. Device images provided by Diagnostic Green®. Reproduced with permission from ©2025 Diagnostic Green®. NIR: near-infrared

The procedure was performed under general anesthesia, utilizing the IC-Flow 2^TM^ (Diagnostic Green LTD; Munich, Germany) device with ICG-mediated NIR fluorescence imaging. Surgical intervention was conducted in a stepwise approach. First, a radical tumor resection was performed with standard technique via transoral laser microsurgery (TLM) (Figure 1) [6]. Following complete tumor resection with clear oncologic margins to reduce the risk of local recurrence, an anterolateral thigh (ALT) flap was harvested from the patient’s left thigh (Figure 2). Prior to flap anastomosis, an initial dose of 3 mL (7.5 mg) of ICG was administered intravenously to assess perfusion and overall viability. The flap was meticulously anastomosed to the ipsilateral facial artery and vein using microsurgical techniques to ensure optimal vascular patency. Subsequently, a second 3 mL (7.5 mg) dose of ICG was administered, and perfusion was assessed 45 seconds post-injection using the IC-Flow 2^TM^ device. Figure 3 summarizes the operative approach. Imagery feedback demonstrated uniform and robust perfusion throughout the flap, confirming successful vascularization and tissue viability. The real-time assessment provided the surgical team with objective intraoperative data, reinforcing surgical conduct in the reconstructive outcome and allowing for immediate corrective measures if perfusion deficits were identified (Video 1 and Figure 4). The ability to dynamically evaluate tissue vitality intraoperatively served to minimize the risk of postoperative complications, particularly flap necrosis. The procedure was completed without complications, and the patient was transferred to the surgical intensive care unit for continued close monitoring during the first 48 hours.

**Video 1:** FGS Intraoperative ALT flap during anastomosis. Notice the contrast between flap edges and stitches, denoting preserved vascularity and overall tissue vitality following the inset. FGS: fluorescence-guided surgery, ALT: anterolateral thigh

Postoperative care

**Figure 5:**
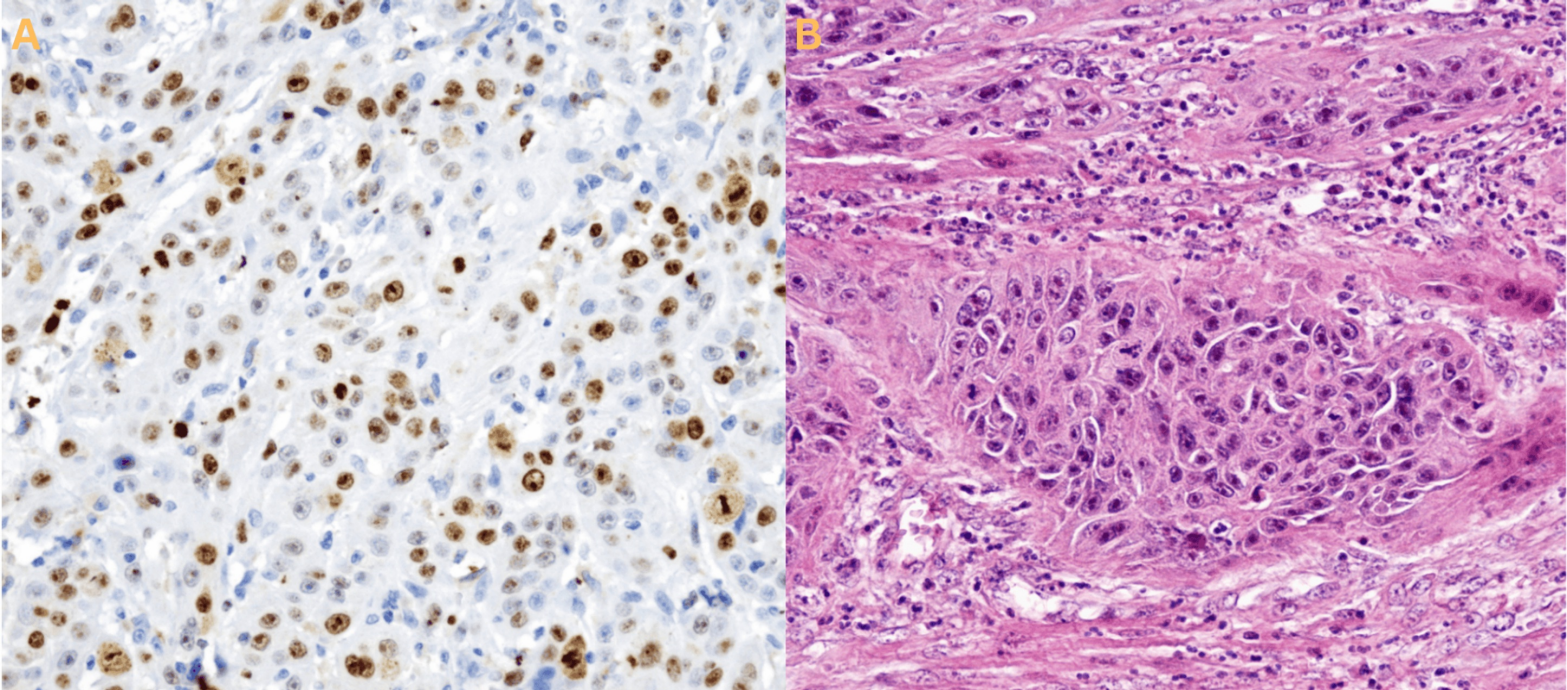
Histopathology Microscopic histopathology of the resected squamous cell carcinoma. (A) Moderately differentiated OPSCC with an intense reaction to antibody immuno-marking. (B) Moderately differentiated OPSCC with H&E staining. OPSCC: oropharyngeal squamous cell carcinoma, H&E: hematoxylin and eosin

Histopathological analysis of the surgical specimen revealed a moderately differentiated squamous cell carcinoma (grade 2: T2N0M0 (TNM Classification for a non-HPV-mediated OPSCC)), measuring 8 x 6 x 4.5 cm (Figure 5). Perineural and lymphovascular invasion were identified on microscopic examination, indicative of aggressive tumorigenicity and supporting the need for adjuvant therapy. All resection margins were negative for malignancy, with no evidence of residual carcinoma, confirming the adequacy of surgical clearance. Flap viability was assessed through serial clinical evaluations and hand-held Doppler ultrasonography to identify early signs of vascular compromise, including arterial or venous thrombosis. The postoperative course was uneventful, with no evidence of flap necrosis, wound dehiscence, or systemic complications. The patient was discharged home on postoperative day 10 with detailed wound care instructions and a structured follow-up regimen in place. In alignment with the multidisciplinary treatment plan, she was referred for adjuvant outpatient RT to mitigate the risk of locoregional recurrence.

## Discussion

The integration of ICG-based NIR fluorescence imaging for intraoperative perfusion assessment marks a significant advancement in surgical oncology. By providing real-time, augmented visualization of tissue vascularity, this technology enables precise decision-making during intraoperative procedures. In the present case, FGS was successfully utilized in the surgical management of an OPSCC radical resection in a high-risk elderly patient, enhancing operative precision and minimizing postoperative complications. Overall, the synergy of standard surgical techniques and improved optics continues to demonstrate its leading potential in managing complex surgical scenarios. The interplay of FGS in surgical oncology holds significant potential to inform clinical guidelines and improve therapeutic outcomes for patients seeking definitive solutions without compromising their quality of life.

Current clinical guidelines-based practice places surgery and RT as the two principal modalities used in the treatment of patients with OPSCC. Per the ASCO guidelines on OPSCC, surgical resection or RT can be the primary approach if the tumor is small and not invasive. Nevertheless, in the setting of invasive disease or larger tumors, a combination strategy is encouraged [7]. In a comparative study examining the association between primary treatment modality for advanced-stage OPSCC and survival outcomes, primary treatment with definitive chemoradiotherapy (CRT) versus upfront surgical treatment was not found to be significantly associated with an increased risk of death during the study period, even after adjusting for baseline factors associated with prognosis [8]. Conversely, a national multicenter cohort study of 726 patients with OPSCC found that those who underwent primary surgery had an increased risk of short-term dysphagia compared with those receiving primary RT or CRT. The patients in the RT/RCT group had an increased risk of short- and long-term gastrostomy tube dependence and worse five-year overall survival compared with those in the primary surgical group [9]. Altogether, the five-year survival rate in OPSCC is approximately 60%. This prognosis varies and is directly correlated with its etiology and therapeutic approach. Notably, HPV-positive OPSCC shows a better prognosis and increased response to treatment compared to HPV-negative OPSCC. This finding, however, has been linked to a more favorable tumor biology and a healthier, younger patient population [10].

For the surgical management of OPSCC, minimally invasive procedures are fundamental. This approach de-intensifies treatment by significantly reducing the long-term toxicity and treatment-related morbidity associated with RT/RCT. Primary surgery also enables accurate pathological staging and helps guide postoperative strategies. The introduction of TLM has revolutionized the surgical landscape, demonstrating excellent functional outcomes. While growing evidence suggests that TLM offers oncologic outcomes comparable to alternative therapies, the debate over definitive approaches continues [11]. Nonetheless, for T1/T2N0 OPSCC, primary surgery is a highly effective therapeutic strategy, given its association with improved overall survival and the high incidence of multiple primary cancers [12]. It is important to note that the potential for functional morbidity is inherently linked with primary surgery and necessitates careful consideration of the unequivocal need for subsequent reconstruction. Thigh flaps, particularly ALT flaps, are commonly and effectively used for reconstructing defects in the head and neck, including those resulting from OPSCC surgery. These flaps offer extensive defect coverage, good vascularity, adequate volume, relatively concealed scarring, and preservation of functional and aesthetic outcomes. FFR may be used for T1/T2 OPSCCs. At one-year follow-up, FFR has shown comparable functional outcomes irrespective of anatomic barriers [13,14].

The patient described in this case was diagnosed with HPV-negative, moderately differentiated stage II OPSCC. The operative strategy was pursued despite the high-risk comorbidities present at the time of surgery. Additionally, postoperative planning was crucial, given that the staging strongly recommended adjuvant therapy. For reconstruction, an ALT flap was deemed the best option compared to a sliding flap. Due to the nature of the reconstruction strategy, the intervention warranted preemptive measures against potential complications, primarily fixation failure secondary to flap necrosis [15]. The use of FGS via the IC-Flow 2^TM^ device allowed immediate assessment of perfusion dynamics during FFR, facilitating timely interventions such as revision of vascular anastomoses or the selection of alternative reconstructive strategies. Unlike conventional, subjective evaluation methods, ICG imaging provides improved metrics of tissue viability, which is critical in patients with compromised vascular status [5]. Recent literature supports the role of FGS in reducing flap failure rates in head and neck reconstruction, with reported sensitivity and specificity for detecting vascular insufficiency exceeding 90% in select series [4,5]. Moreover, FGS with ICG facilitates real-time tumor-margin delineation, improving margin clearance rates and progression-free survival, as evidenced by the negative-margin histopathologic analysis of this case. Although the use of ICG has been previously described for intraoperative FFR assessment, the present case highlights the transformative role of this technique in head and neck oncologic surgery. While limitations included the need for specialized equipment and training, as well as patient-specific variables such as hepatic dysfunction or hypersensitivity reactions, no adverse events were encountered in this case. The technique was readily incorporated into the operative workflow, further underscoring its practical utility and potential to improve outcomes in complex oncologic reconstruction.

## Conclusions

This case highlights the utility of advanced perfusion monitoring in oncologic surgery, particularly in OPSCC requiring microvascular reconstruction. The favorable outcome in a high-risk patient demonstrates the potential of ICG-fluorescence imaging to support intraoperative decision-making and enhance surgical safety. Further prospective studies with larger patient cohorts are needed to establish the efficacy, cost-effectiveness, and long-term safety of this approach. Standardized protocols and broader implementation, including in resource-limited settings, will be critical to optimizing its clinical adoption.
